# Spin resolved topological bulk state in acoustics

**DOI:** 10.1038/s41598-024-53226-6

**Published:** 2024-02-08

**Authors:** Mustahseen M. Indaleeb, Sourav Banerjee

**Affiliations:** Integrated Material Assessment and Predictive Simulation Laboratory (i-MAPS), Department of Mechanical Engineering, University of South Carolina, Columbia, SC 29208 USA

**Keywords:** Acoustics, Mechanical engineering

## Abstract

Extremely rare t*opologically protected* acoustic energy sink is presented in this article. Acoustic topological phenomena are generally described using quantum anomalous hall effects (QAHE), quantum valley hall effects (QVHE), and quantum spin hall effects (QSHE) where spin orbit coupling is predominant. Topological edge states are demonstrated by bulk-boundary distinction when the bulk is insulated. In this article topological acoustic conductor and its phenomena are theoretically demonstrated where the boundaries are insulated. This is exactly opposite to the behavior of a topological acoustic insulator. Phenomena presented in this article could not be explained by any of the trio Quantum Hall effects. To explain the phenomenon phononic crystals or PnCs were designed to obtain accidental triple degeneracies, resulting a Dirac-like cone at the Γ point ($\overrightarrow{k}=0$). The phenomenon is microarchitecture and microrotation field independent. Here time reversal symmetry or the space inversion symmetry is not broken, and the degenerated ‘Deaf band’ dominates the local dispersion with a syncline top band. This scenario results in continuously changing ‘up spin’ and ‘down spin’ of the wave energy in the media and remain trapped without specific preferential direction of wave transport. The spin was found to generate the spin angular momentum, causing the switching in geometric phase from $0-2\pi$ in cyclic pattern, keeping the energy trapped inside the bulk media.

## Introduction

Bulk-boundary distinction of edge states and modulation of counter intuitive wave propagation were widely studied in the fields of electromagnetism and acoustics. Innovative methods for controlling the waves resulted in the discovery of topological phenomena. The advancement of topological research in the field of acoustics is somewhat challenged compared to the field of electromagnetics. This is due to the complexity of the practical implementation of the spin degrees of freedom. Topological edge states and the topological bulk states are distinct from each other. Topological insulators are created when the boundaries are conductive (i.e., energy propagates along the boundary). Thus vice versa topological conductor can be created when only the bulk material is conductive, keeping the edge state partially insulated. In this article such acoustic topological phenomena are theoretically demonstrated and explained. This is exactly opposite to the behavior of a topological acoustic insulator, and we call it topological acoustic conductor. Phenomena presented in this article could neither be explained by the Quantum trio Hall effects, nor by the spin orbit interaction (SOI) of acoustic field in an articulated meta structure where micro-rotational field is created by virtue of the design of the structure. Here first the quantum trio effect and then the SOI are discussed in the following paragraphs.

### Quantum trio

Topological insulators were originally proposed in quantum systems^1^. The topological insulators were designed^2–9^ for photonic systems using the phenomena called Quantum Anomalous Hall effect (QAHE). QAHE required an external magnetic field to break time reversal symmetry. Following the electromagnetic waves and its mechanisms, analogous quantum Hall effect in acoustics was hard to achieve. This is because there is no equivalent magnetic field that exists in acoustics. Instead of finding an equivalent magnetic field, researchers attempted to mimic the behavior generated by the magnetic field. The behavior is the spin polarization^10–13^. Such spin polarization was first realized in 2015^14^ by theoretically injecting external flow fields to break the time reversal symmetry. Later the acoustic Chern insulator was experimentally validated^15^. Requirement of additional energy to create an internal flow field was considered a drawback of QAHE in acoustics. With further progress, utilizing the pseudospin states, many feasible designs of flow-free acoustic topological insulators^16–18^ are proposed. Robust one-way propagation based on topological edge states were demonstrated in several metamaterials made of different types of phononic crystals (PnCs).

In quantum spin hall effect (QSHE)^19^ acoustic topological insulators have a double Dirac degeneracy at the center of the Brillouin zone where topological phase inversion is inevitable^17,20^. The pseudospin states at the double Dirac point form a topological edge state and could be programable^21^. Creating a bulk-boundary distinction, immune to back scattering, one way wave propagates along the interface between ordinary PnCs (trivial state) and topological PnCs (non-trivial state). In quantum valley hall effect (QVHE)^22–26^, graphene like hexagonal unit cells, degeneracy of two locally linear bands forms a Dirac cone at the Brillouin boundary. In the case of QVHE, a pair of separated valley vortex states are formed. These valley vortex states have opposite chirality and can also realize topological edge states. Edge state is along the interface called the domain wall between two domains made of similar but opposite orientation of the unit cells. Despite different requirements of excitation, valley Hall PnCs demonstrate the ability to hold robust one-way propagation.

It is to be noted that the QAHE breaks the Time reversal symmetry, whereas in QSHE and QVHE the time reversal symmetry is protected. In QSHE two states with opposite spin polarization resemble two copies of the quantum Hall effect. The spins are correlated to the direction of propagation of the wave and opposite spin means counterpropagating at a given edge. In acoustics however, traditional spins like in quantum mechanics (fermions) are not present. Spin-1/2 fermions are said to be impossible to achieve. However, phonons may have full integer spin, simulate spin-1 boson like particles. A fundamental difference between the spin-1/2 fermions and spin-1 photon/phonons are the Kramers doublets^27^ and is a mandatory condition for QSHE. This is also absent in acoustics. However, by understanding the physics of QSHE and its prerequisites, it is realized that a metamaterial and in its unit cell or a PnCs must have more degrees of freedom to simulate the spin through inducing microrotational fields. Then Kramers doublet could be artificially created in acoustics as a pair of pseudospin states like electrons. Hence, in acoustics, due to its longitudinal curl-free nature the waveguide modes for QSHE are traditionally described with *spin-like modes* (*pseudospin*) rather than real Spin Angular Momentum (SAM). The equivalence of acoustic pseudospin and real SAM are thought to be absent in acoustics.

### Spin orbit interaction (SOI)

Beyond quantum trio examples it is realized that by absorbing the acoustic SAM, a small particle will receive a torque proportional to $s$. Total angular momentum (TAM) in acoustics is divided into two parts, orbital angular momentum (OAM) and SAM. OAMs are due to the energy vortices, or the rotation of the energy flux generated from spatial phase gradient synonymous with the scalar degrees of freedom. SAM, on the other hand, is developed due to the rotation of the polarized velocity field vectors. It is shown that the spin–orbit interaction (SOI) between OAM and SAM is possible in acoustics^28–32^. Creating nonhomogeneous metamaterial with inherent microrotation of acoustic field researchers demonstrated the spin–orbit interaction (SOI). Such interaction must be absent for longitudinal waves in acoustics^30^. But if the acoustic energy demonstrates any non-zero acoustic spin density, then spatially the acoustic energy must support the spin state of the wave generated from longitudinal and transverse wave modes^31,32^. It is further shown that the spin could be transported^29^ in articulated waveguide through locally rotational velocity field. Later by exploiting both the velocity and microrotation field two types of SOI in momentum and real space were demonstrated^28^. It is to be noted that in these studies, it is required to mechanically create the rotational fields by virtue of creating articulated structure in a specific pattern.

In this article without creating such articulated structures or by mechanically creating microrotation of the vector field, a deaf band based naturally occurring spin state is demonstrated. Periodic metamaterials made of PnCs were used to demonstrate that the spin state (a) is frequency dependent, (b) generated due to abnormal polarity, (c) only depends on the deaf band and is also (d) microstructure independent. Through numerical experiments we theoretically show that such spin state near deaf band is solely responsible for topological bulk state. One way of verifying the existence of spin in bulk is the spatial integration of the acoustic spin density for a localized wave mode. Such integration must result in zero to agree with spin-0 nature of the phonons. Beyond nonzero spin we show that a unique spatial distribution of the ‘up-spin’ and ‘down-spin’ state is generated. An intrinsic acoustic SAM of an acoustic field is obtainable by a velocity field with rotational polarized properties despite being curl-free. The SAM in acoustic wave can be expressed as:

1 $$s = \frac{\rho }{2\omega }Im\left[{v}^{*}\times v\right]$$

where $\rho$ is the mass density, $\omega$ is the frequency of interest, $v$ is the velocity of particle vibration and ${v}^{*}$ is the conjugate of $v$. Further details can be found in the supplementary material.

### Abnormal polarity

It was found^33,34^ that sometimes due to effective anisotropy of a metamaterial longitudinal wave modes may manifest orthogonal polarization (polarization orthogonal to the direction of propagation) and transverse wave modes may manifest longitudinal polarization (polarization along the direction of wave propagation). These are anomalous polarization characterization of the wave modes. They can be effectively demonstrated when the wavevector moves along a certain equifrequency contour and transitions from one wave mode to another wave mode present at the same frequency. Although acoustic waves do not have transverse wave modes, polarization of a specific band may manifest transverse polarization. If such a state exists, superposition of these phonons is bound to create SAM. In this article it is proved that SAM is solely responsible for creating the bulk energy sink in a topological conductor.

### Topological blackhole

Unlike QSHE where two Dirac-like cones are degenerated at the center of the Brillouin zone, there are other situations where an almost a flat band called ‘Deaf band’ with antisymmetric mode shape is trapped in between a Dirac cone at the center of the Brillouin zone. Earlier in several articles^35–38^, exploiting the behavior of triply degenerated Dirac-like cone, several unique phenomena and applications are presented in detail. However, in this article a recently discovered novel phenomenon is presented near the Dirac frequency, when the Dirca-like cone degeneracy is lifted. The Three-band degeneracy is composed of a top band, a deaf band, and a bottom band. Dirac-like cone is formed when these three bands are degenerated. It is found that there is no instance along the frequency scale, when all the three bands are separated. Either top or the bottom band always remain degenerated with the deaf band. Tracking their behavior when at least one band is lifted from the degeneracy at a specific frequency, the topological phenomena emerge.

Here beyond the Quantum trio, a deaf band-based physics of a topological phenomenon is discussed in acoustics. A spin controlled bulk conduction waveguide, keeping the edge state insulated, is demonstrated. *Without any intricate artificial design, acoustic spin state could be naturally manifested which is shown in this article.* Periodic phononic crystals, called PnCs, are utilized in this acoustic design. In these cases, even when the spatial symmetry is broken, time reversal symmetry is protected. This phenomenon is independent of the shape of the PnCs as long as the degeneracy criteria between the deaf band and an upper band is satisfied. Encouraged by the deaf band-based energy modulation, topological bulk conduction is achieved with negligible leakage. Hence, this phenomenon is named ‘Topological Blackhole’ (TBH). Please note that the topological black hole is not to be confused with the acoustic black hole, previously reported in the literature^39–43^.

## Materials and method

**Figure 1:**
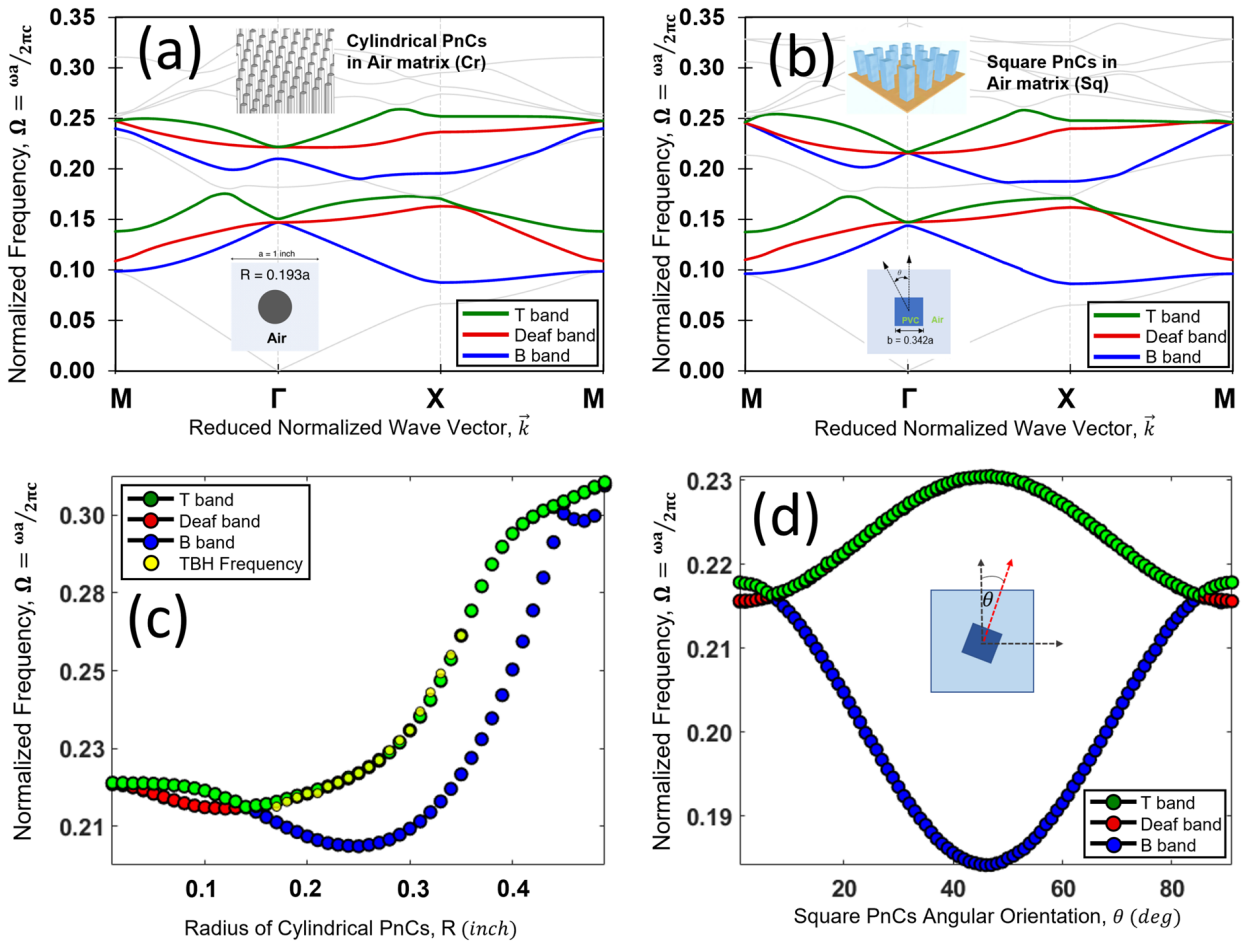
(**a**) Band dispersion diagram for cylindrical PnCs (Cr) arranged in square lattice in air media. The unit cell side, a = 1 inch, with a filling fraction ${F}_{f}$ = 0.1169 with Cr crystals inclusion of radius, r = 0.193a. Two frequency regions of interests with three bands each, named as Top (T) Band, Deaf (D) Band and Bottom (B) band, are plotted in colors (Green, red and blue respectively). Unit cell and PnCs distribution are shown as inset. (**b**) Band dispersion diagram for square PnCs (Sq) arranged in square lattice in air media. Like (**a**), the filling fraction of the PnCs inclusion has been kept consistent (${F}_{f}$ = 0.1169) in this case. (**c**) Shows how the normalized frequency eigen value of the dispersion bands of interest changes at the $\Gamma$ point with varying size of the Cr diameter i.e. changing the ${F}_{f}$. (**d**) Similarly shows how the normalized frequency eigen value of the dispersion bands of interest changes at the $\Gamma$ point with the increasing angle of rotation (angle of PnCs w.r.t angle of incidence) of the square PnCs from $\theta = {0}^{^\circ } to \,{90}^{^\circ }$.

**Figure 2:**
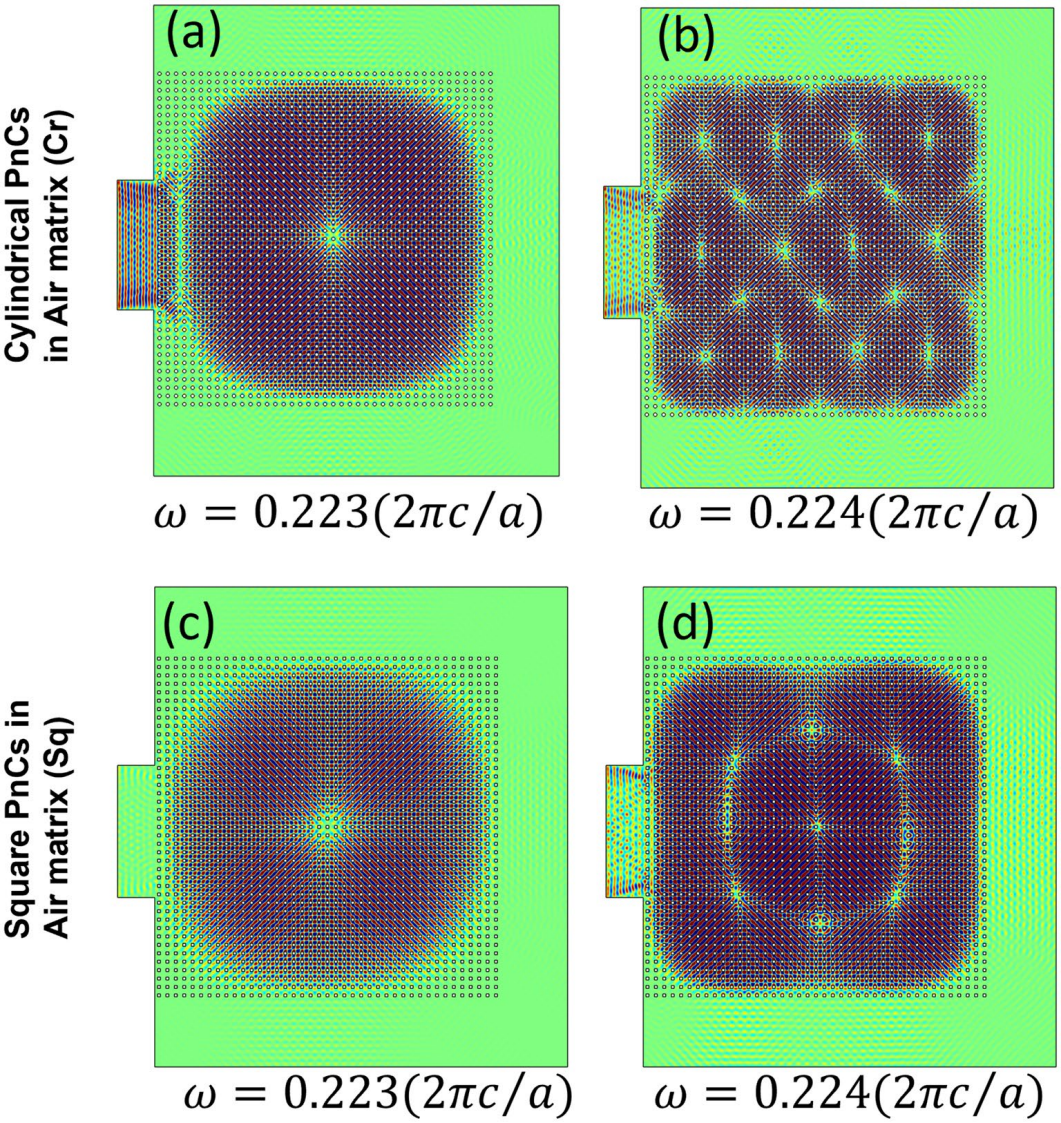
(**a**) and (**b**) Spatial distribution of absolute acoustic pressure inside a 41 × 41 matrix of cylindrical PnCs with r = 0.193a. The boundary of a waveguide designed at the left of the PnC matrix is actuated at the frequency ($\omega =0.223\left(2\pi c/a\right) and \omega =0.224\left(2\pi c/a\right) respectively$) and the energy seamlessly remains contained/trapped within the periodic media, keeping the edge topologically protected. Such a phenomenon is extremely unique, where an energy sink develops within the periodic media, keeping most of the energy trapped in the bulk. Increasing the actuation frequency results in multi-modal energy trap keeping the edge state almost unperturbed or intact. No or almost negligible leakage through the edge is observed, making it a perfect energy sink, or an acoustic topological blackhole (TBH). (**c**) and (**d**) depict similar phenomena using square PnCs (b = 0.342a). For both the cases, the topological edge state remains undisturbed.

First, two types of PnCs are considered namely cylindrical rods (Cr) and square rods (Sq) (Fig. 1a,b insets). The PnCs made of polyvinyl chloride (PVC) were repeated in a square periodic media of air matrix having two-dimensional periodicity. Fraction of the area of the phononic crystal made of PVC in a square unit cell made of air is defined as the filling fraction (${F}_{f}$). Applying block-wave theory of a periodic media the dispersion equation was formulated^35–38^. Solving the appropriate eigen value problem (refer supplementary material section S.1) for two different PnCs wave dispersion plots were generated. Figure 1a shows the band structure for a periodic media with a unit cell made of Cr PVC rod in air media. Similarly, Fig. 1b shows the band structure of a unit cell with a Sq PVC rod in air media. In both cases with Cr and Sq crystals the filling fraction (${F}_{f}$) of the unit cells were kept constant, ${F}_{f}$=0.1169. Next how the Dirca-like cone behavior changes with varying geometrical parameters were studied. Figure 1c shows how the frequency eigen value changes at the $\Gamma$ point with varying size of the Cr diameter changing the ${F}_{f}$. Although the ${F}_{f}$ are changed, the Cr geometry is still symmetric. This is not same when the Sq elements are rotated keeping the ${F}_{f}$ constant. Figure 1d shows how the Frequency eigen values change at the $\Gamma$ point with varying angle of rotation of the Sq element. At ${F}_{f}$=0.0623 the equivalent Cr diameter equal to 0.1408a (a is the lattice constant) the Dirac-like cone is formed in Fig. 1c. Above this cutoff ${F}_{f}$ the deaf band is degenerated with a syncline top band. If the complete wave number domain over the entire first Brillouin zone is considered, similar negative curvature is also true for the deaf band. However, the anti-symmetric deaf band is almost flat near the center of the Brillouin zone. Figure 1d shows at an angle of rotation $\theta ={\pm 7}^{o}$ the Sq elements created the Dirac-like cone when the spatial inversion symmetry is broken. For $\theta$ between $\pm 7^{ \circ }$ and $\pm 83^{ \circ }$ the deaf band remains degenerated with the top band. Near the close proximality to the Dirac-like cone both the top band and the deaf band are syncline in nature, manifest curvatures with increasing wave number along $\Gamma -{\text{X}}$ and $\Gamma -{\text{M}}$ over the first Brillouin zone. In both cases the above-mentioned TBH criteria is satisfied. Next, using the finite element commercial software, wave fields were generated in the megastructure presented in this study composed of both Cr and Sq crystals. All analysis in this article was performed in COMSOL Multiphysics software^44^. Please note that no solid hard boundary was used in the simulations presented in this article. In addition, please note that no non-reflection boundary or absorbing boundary conditions were used in the simulation presented in this article. Figure 2a,b, shows the contained energy sink and the wave field inside a square meta structure made of 41 × 41 Cr crystals. At two different normalized frequencies close to Dirac frequency Cr crystals satisfying the TBH requirement and generated the energy sink only inside the bulk and the edge state is unperturbed with minimum leakage. Figure 2c,d, shows the contained energy sink and the wave field inside a square meta structure made of 41 × 41 Sq crystals. At two different normalized frequencies (same as Cr crystals) Sq crystals satisfying the TBH requirements within the TBH zone, an energy sink is generated only inside the bulk and the edge state is unperturbed with minimum leakage. It is shown that with and without spatial symmetry the TBH phenomena is persistent under certain conditions.

To prove that this topological state is protected against the space inversion symmetry (i.e. when the space inversion symmetry is broken by rotating the Sq crystals), Figure S1a in supplementary document shows the energy sink in a similar square meta structure made of 41 × 41 Sq crystals when the crystals are rotated at $\theta ={\pm 1}^{o}$, $\theta ={\pm 4}^{o}$, $\theta ={\pm 6}^{o}$ and $\theta ={\pm 10}^{o}$, respectively. Although not shown the phenomena remains persistent with rotation $\theta ={\pm 15}^{o}$. Fig. S1b identify the zone that carries the TBH phenomena. Collecting the frequency of occurrence of the TBH phenomena at each angle of rotation Fig. S1c shows that they are not equal to the deaf band frequencies, nor it is equal to the top band frequency. Please note that the frequency of occurrences of the TBH phenomena is following the syncline curvature of the deaf band at an offset near the top band over the parameter space (here rotational angle). Thus, it is concluded that the TBH phenomena is not solely due to the antisymmetric deaf band which may appear to be true at first instance. But it is an effect that is superposition of the top band and deaf band, which played a role through their abnormal polarity discussed in results.

**Figure 3:**
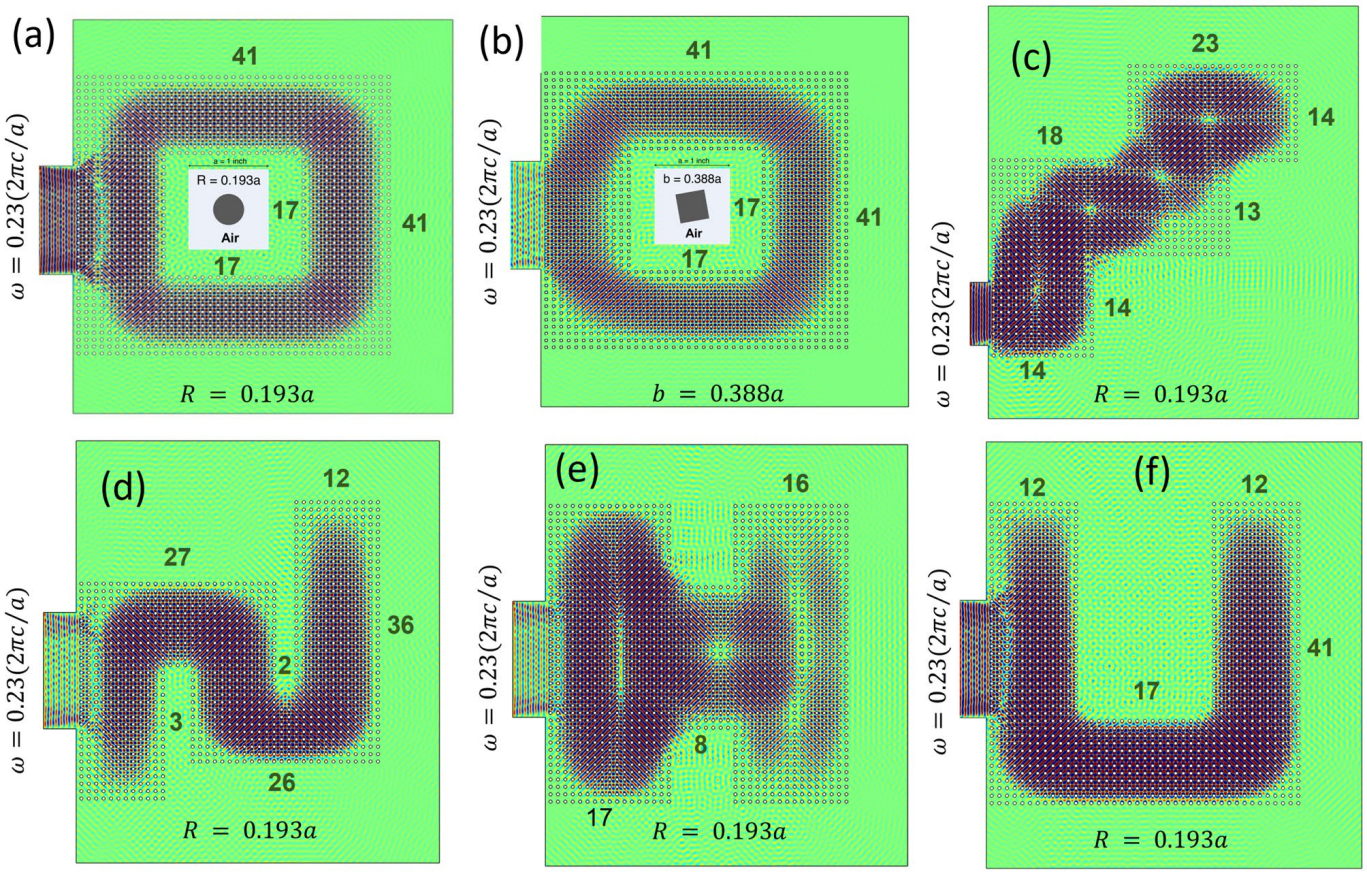
Robustness of the TBH phenomenon is confirmed here in. Varying the waveguide shape by changing the PnCs distribution it was observed that the TBH remains intact and undisturbed, keeping the energy sink within. TBH is visible in (**a**) and (**b**) Square ring-shaped matrix using cylindrical (Cr) and square (Sq) PnCs respectively, further it shows (**c**) the energy sink in a step shaped wave guide, (**d**) in a S-shaped wave guide, (**e**) a H-shaped meta structure, and (**f**) a U-shaped matrix. The number of unit cells used to compose the structure are shown in respective images.

Figure 3 shows the topologically protected wave sink where different shapes of the meta structure with Cr or Sq crystals are used. Figure 3a shows the wave field in a meta structure made of similar 41 × 41 Cr crystal but 17 × 17 elements from the middle portion of the slab was removed to make an annular ring. Figure 3b shows the wave field in a meta structure made of similar 41 × 41 Sq crystal but 17 × 17 elements from the middle portion of the slab was removed to make an annular ring. Figure 3c is created like a stair made of Cr crystals using the unit cells mentioned in the figure. Figure 3d shows the field in an S shaped structure. The unique manifestation of bulk energy sink is evident in two other shapes presented in Fig. 3e,f.

## Results and discussions

### Condition for TBH with the state of degeneracies at dirac-like cones

It was found that when the curvature of the upper band is syncline in nature, having negative curvatures over the wave number domain, but the deaf band is almost flat near the center of the Brillouin zone, the TBH phenomena prevail. Additionally, although the deaf band is almost flat near the center of the Brillouin zone, it might manifest a curvature with increasing wave number along $\Gamma -{\text{X}}$ and $\Gamma -{\text{M}}$. Type of such curvature must match with the top band curvature. That’s means the deaf band must also be syncline with slowly varying negative curvature compared to the top band. When an anticline deaf band and an anticline bottom band with positive curvature over wave number domain are degenerated, TBH criteria is not satisfied. An additional interesting phenomenon was observed that a range of governing parametric may satisfy TBH state when the deaf band eigen frequency curve has a negative curvature with respect to the parametric space. It is realized that a doubly degenerated state formed at the Г point may manifest TBH under certain circumstances.

All these states are linked to the spin states discussed as follows. Despite band diagram and the syncline requirements, the TBH phenomena can be explained using the polarity of the wave modes and how does they contribute to the local and global spin. It was found that the polarization of two energy modes degenerated at the Г point needs to be orthogonal to each other to achieve TBH. Such mismatched polarity results in an ambiguity within the energy propagating through the PnCs, resulting in alternate spinning to allow the energy to be trapped within. Changing the shape and size of the waveguide does not destroy the TBH, confirming high robustness of the system. Thus, TBH is topologically protected. Regardless of any defects inside the meta structure made of PnCs array, the topological behavior persists when excited at the doubly degenerated frequency. Hence, the energy trapping is independent of the impurities within the metamaterial made of PnCs.

### Anomalous polarity and SAM

Anomalous polarity and its effect on wave propagation can be found in Ref^33,34^. Wave polarization in acoustic waves is associated with the longitudinal wave modes. Acoustics in fluids (liquid or gas) do not support transverse waves, and thus the transverse wave modes do not exist. However, in TBH it is found that the polarization of the acoustic wave field, and its spin plays a critical role. Although acoustic phonons do not support spin, the polarized vector field at the state of TBH is found to defy this notion. Global velocity under TBH state has an inherent polarity that changes over time. The polarized velocity continuously rotates first in clockwise and then anticlockwise directions. Global spin of the acoustic field is calculated and is found to be counterintuitive. Total wave field from time-domain analysis in the meta structure with Sq crystals ($b=0.342a$, ${\theta =9.7}^{^\circ }$) is integrated to find the resultant velocity vector at any instant. Although not shown, similar is true for meta structure made of Cr crystals. While entering the metamaterial made with Cr or Sq crystal matrix, acoustic phonons associated with the deaf band and the top band naturally initiates the local micro-spin (not by creating specific articulated metamaterials^28–32^), which globally forms a wave sink, where the wave appeared to be trapped inside a blackhole without any escape. This phenomenon is topologically protected.

**Figure 4:**
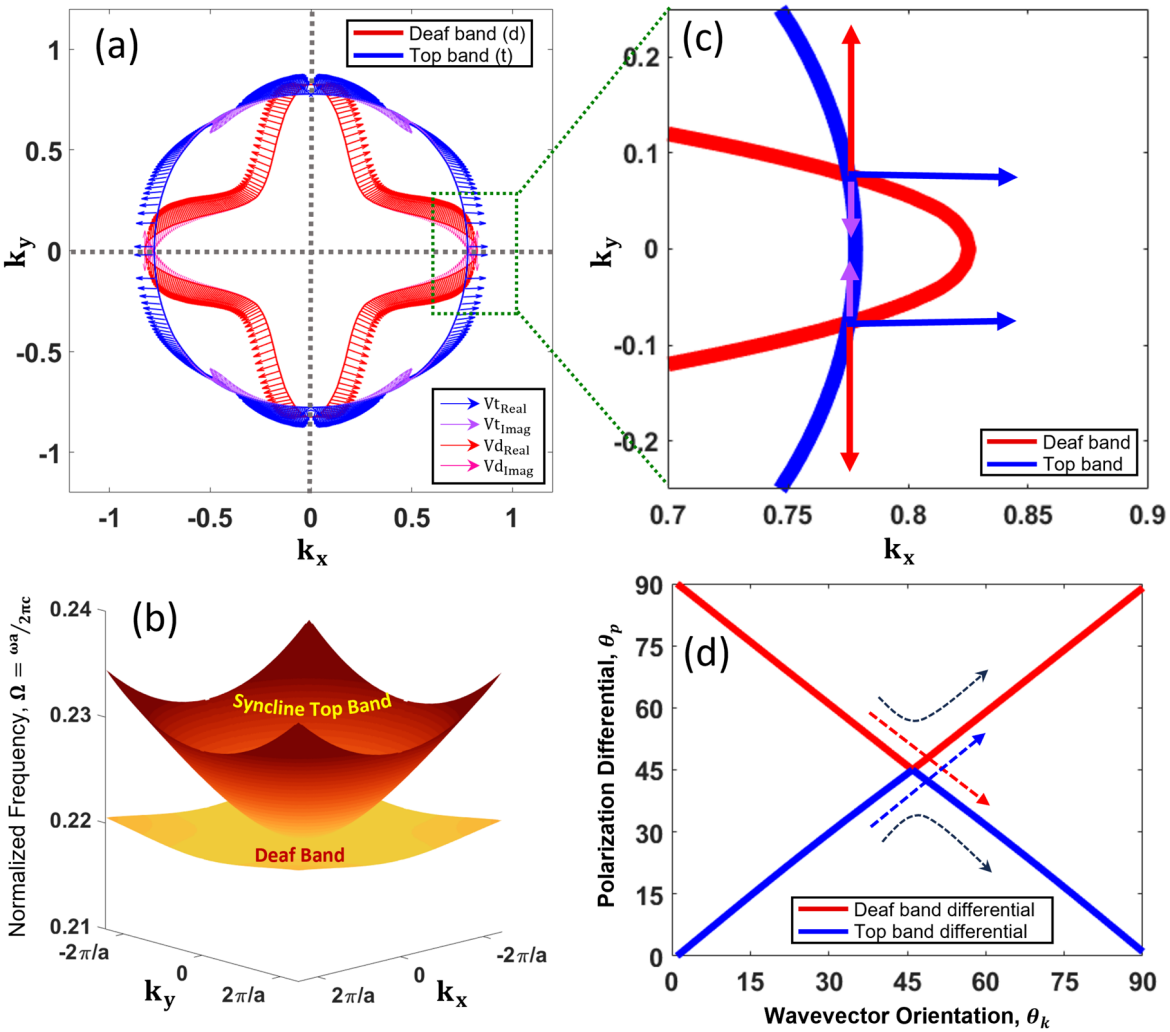
(**a**) Polarization characteristics vectors (both real and imaginary) of normalized wave velocity (${V}_{t}$) in reciprocal space are shown for both Deaf band (red) and Top band (blue), (**b**) Equifrequency contour of Deaf band and Top band, (**c**) Polarization anomaly at the intercepted junction of both the bands of two EFS modes, demonstrating the origin of the phenomena from the anomalous orthogonal polarity. (**d**) Anomalous polarity is demonstrated plotting the polarity differentials from the polarization orientation plot of the Top band and the Deaf band with respect to the wave vector angle on x–y plane with respect to $\Gamma -X$ direction. Only ${0}^{o} to {90}^{o}$ orientations are shown.

As discussed earlier, the TBH frequency is very close to the degenerated frequency of the deaf band and the top band. Near the Г point of the BZ, cross section of the normalized equifrequency contours (EFC) at TBH frequency are plotted in Fig. 4a. The red EFC belongs to the deaf band and the blue EFC belongs to the top band from the band structure. Figure 4b shows the equifrequency surfaces (EFS) of both the bands (deaf and top). Figure 4a also shows the polarity $({\theta }_{p})$ of the real and imaginary part of the velocity vectors. The polarization vectors are illustrated with arrows at each wavevector direction at an interval of ${\theta }_{k} = {1}^{o}$. If the polarities at the intercepts of normalized EFCs are not aligned, a non-trivial anomalous polarity emerges. Figure 4c portrays the polarity ambiguity for the deaf band and the top band, having orthogonal polarities at the junction of interception. However, with close observation (Fig. 4a) it can be found that the polarity of the real part of the velocity of the deaf band and the top band are always orthogonal for all possible directions of wave vectors. Next, to characterize the wave polarity with respect to the direction of the wave vector $\mathbf{k}$, a new parameter called polarity differential (${\theta }_{pd}$) is created. The polarity differential for each wave mode (top, deaf) is calculated by subtracting the wave propagation angle (${\theta }_{k}$) (i.e. the angle made by the wave vector $\mathbf{k}$ with respect to a reference plane) from the angle of the direction of the particle motion or simply the polarization angle (${\theta }_{p}$) (obtained from the real polarization vectors of the respective wave modes) with respect to the same reference plane. Figure 4d illustrates the change of ${\theta }_{p}$ of the two bands over one quadrant. Polarity differential seems to intercept at ${\theta }_{k} = {45}^{{\text{o}}}$. Such merging of polarity validates anomalous polarization. How this anomalous polarity contributed to the local SAM density as discussed in the following sections.

### Local analysis for SAM

To visualize the force field instantaneous acceleration was calculated. After numerically solving the wave field at TBH frequency, the local instantaneous accelerations, ${a}_{1}({x}_{j},t)$ and ${a}_{2}({x}_{j},t)$ were collected. The resultant instantaneous acceleration at every spatial point was calculated using the following equation.

2 $$\mathbf{a}({x}_{j},t) = \sqrt{{a}_{1}^{2}({x}_{j},t)+{a}_{2}^{2}({x}_{j},t)}$$

**Figure 5:**
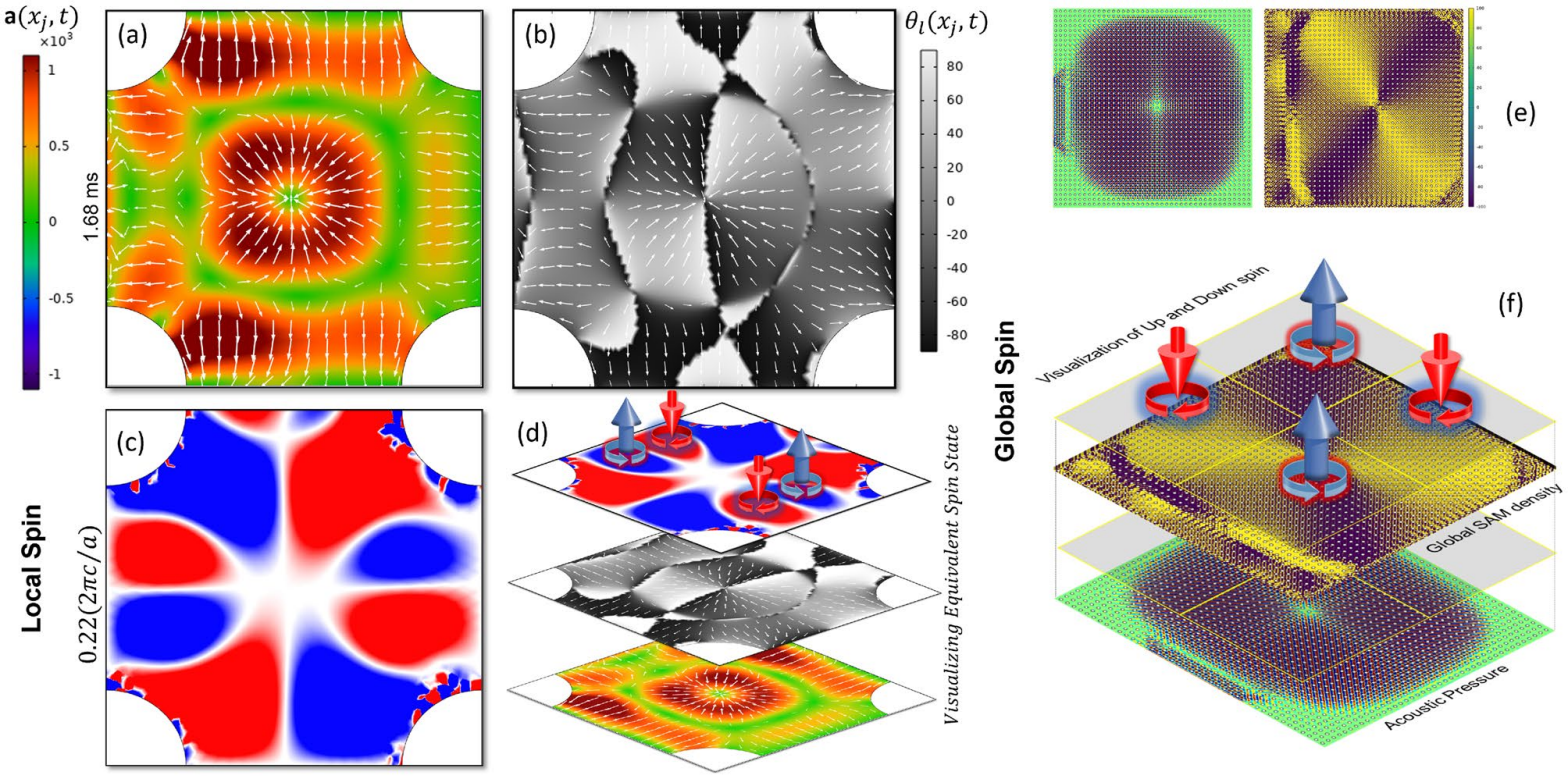
A snapshot in time at after 1.68 ms (**a**) shows the superposition of acceleration vector field and the surface distribution of instantaneous total acoustic acceleration field. It demonstrates how the vector fields fluctuate over time continuously converge at and diverge from the center of the unit cell. (**b**) shows the superposition of the surface map of the polarity of the vector field with the surface distribution of the acceleration vectors with relative magnitude. (**c**) Shows the surface distribution of the local spin angular momentum (SAM) density in a unit cell obtained from frequency analysis at the normalized frequency very close to the TBH phenomena. (**d**) A stacked image shows the vector field and the local SAM density obtained from time domain analysis and frequency domain analysis, respectively at TBH frequency from a unit cell. (**e**) Shows a repeated plot from Fig. 2a and the compared side by side with a spatial distribution of the global SAM density obtained from the same geometry of the crystal orientation. (**f**) A stacked image shows that at the TBH frequency fully stable and self-balanced quadrupolar Spin-Up and Spin-Down state that are activated in the metastructure made of PnCs.

Spatial distribution of the instantaneous phase angle, ${\theta }_{l}({x}_{j},t)= {tan}^{-1}\left(\frac{{a}_{2}({x}_{j},t)}{{a}_{1}({x}_{j},t)}\right)$ was also calculated. Figure 5a shows the instantaneous acceleration after 1.68 milli seconds over a unit cell made of Cr crystals. Figure 5(b) shows the map of instantaneous phase angle ${\theta }_{l}$ (between − 90° to + 90°) over a unit cell after 1.68 milli seconds (refer supplementary material). From Fig. 5b it is evident that the vector field in four quadrants has negative divergence pointing to an energy sink like it is the case for a black hole. Such a state is at the confluence of opposite spin of the vector field. Clockwise (CW) rotation represents spin down ($-1/2$) and counterclockwise (CCW) rotation represents spin up ($+1/2$) of the phonons. Although, the state of the sink at 1.68 milli seconds was selected arbitrarily, such sink state is periodically occurring in a unit cell. To further understand if the spin exists near the TBH frequencies, the local wave velocity components at different frequencies were calculated as ${v}_{1}({x}_{j},\omega )$ and ${v}_{2}({x}_{j},\omega )$. Spatial velocity vector field was realized and the SAM density at every spatial point over several frequencies at an interval of 5 Hz was calculated using the Eq. (3).

3 $${\mathbf{v}}\left( {x_{j} ,\omega } \right) = \left[ {v_{1} \left( {x_{j} ,\omega } \right)v_{1} \left( {x_{j} ,\omega } \right)} \right];\;\;s\left( {x_{j} ,\omega } \right) = \frac{\rho }{2\omega }\langle{\mathbf{v}}\left| {\varvec{\sigma}} \right|{\mathbf{v}}^{{\varvec{T}}}\rangle$$

where $${\varvec{\sigma}}=\left[\begin{array}{cc}0& -i\\ i& 0\end{array}\right]$$ is called the spin operator. The unit of SAM is kg/(m.sec). Figure 5c shows the positive and negative spin states that are present in a local unit cell orthogonal to the cell with wave incidence (refer supplementary materials). A staked image is created for the unit cell in Fig. 5d consists of Fig. 5a–c to explicitly show that the spin state in frequency domain and the energy sink in time domain coexists.

### Global analysis of the spin

Further to explain the global spin state the global wave velocity components at different frequencies were calculated as ${v}_{1}({x}_{j},\omega )$ and ${v}_{2}({x}_{j},\omega )$ in a meta structure made of 41 × 41-unit cells (ref Fig. 2a,c). Next the SAM density at every spatial point over several frequencies at an interval of 5 Hz was calculated using Eq. (3). Differences between the local and global spin state are obtained using coarser and finer elements, respectively. Thus, it helps visualize the multi-scale spin state at TBH. Figure 5e shows the acoustic pressure field and the respective global SAM density side by side at 0.223 normalized frequency where TBH was observed. A staked image is created in Fig. 5f to show the spin up and spin down states that coexists in a quadrupolar fashion. Similar but multipolar states are shown in supplementary materials at other frequencies that demonstrate TBH.

Next to explain the energy sink, integration of the components of the local force density over the entire bulk media (41 × 41 phononic crystals) was obtained with their respective polarity. Surface integral to achieve the integrated net force field was,

4 $$\begin{aligned} {\mathbf{F}}_{{\varvec{g}}} \left( t \right) & = \iint\limits_{{\Gamma }} {\rho {\mathbf{a}}\left( {x_{j} ,t} \right)dx_{j} ;} \\ {\text{F}}_{1} \left( t \right) & = \iint\limits_{{\Gamma }} {\rho a_{1} \left( {x_{j} ,t} \right)dx_{j} };\;{\text{F}}_{2} \left( t \right) = \iint\limits_{{\Gamma }} {\rho a_{2} \left( {x_{j} ,t} \right)dx_{j} ;\;\theta_{g} \left( t \right) = tan^{ - 1} \left( {\frac{{F_{2} }}{{F_{1} }}} \right)} \\ \end{aligned}$$

were, $\Gamma$ indicates the surface integral over the entire domain of the simulation. Global force field ${\mathbf{F}}_{{\varvec{g}}}\left(t\right)$ has its two components ${F}_{1}$ and ${F}_{2}$. The global phase of the wave field at any instant would be ${\theta }_{g}(t)$ which determines the polarity direction of the force field at an instant $t$. At each time step, the global force vector, ${\mathbf{F}}_{{\varvec{g}}}(t)$ and the orientation polarity, ${\theta }_{g}(t)$ were calculated.

### SAM at TBH

**Figure 6:**
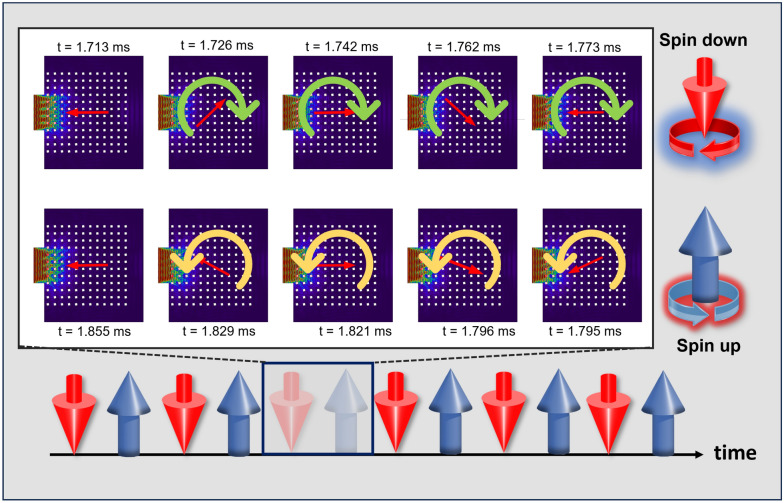
Shows the instantaneous local acoustic velocity field with the calculated instantaneous field polarity. Starting from 1.713 ms, the polarity encounters a CW rotation of 2π at t = 1.773 ms, depicting a *Spin-Down* state in terms of acoustic spin, and then reverse the rotation in CCW starting from t = 1.795 ms by 2π at t = 1.855 ms, depicting a *Spin-Up* state. Such cycle of spin up and down persists with time observed in transient study.

If a particle is arbitrarily placed at any location in an acoustic field and the particle rotates around the particle center, then the particle is said to poses SAM. When the particle rotates around the center of the acoustic field the particle is said to have the OAM which is not the case herein. In this case for TBH we believe the phonons have SAM and do not have any SOI to demonstrate the following behavior. *However, they have a local and global spin coupling as shown in *Fig. 5*.* It was found that the ${\theta }_{g}(t)$ continually changes from $-2\pi$ and $+2\pi$ (or $0-4\pi$) and then again from $+2\pi$ and $-2\pi$ (or $4\pi -8\pi$) while oscillating the spin vector between up and down spin. Figure 6 shows an arbitrarily selected sample segment of this event between $0-2\pi$ and $2\pi -0$ occurred between $t =\sim 1.71 msec$ and $t =\sim 1.85 msec$. It is concluded that the energy is trapped inside the Bulk media due to counter interactive CW (down) and CCW (up) spins. The features of this spin mediation are due to the modal anomalous polarity and switching between the polarity of the deaf band and the top band described in Fig. 4. The counter propagating spins result in the bulk phenomenon of TBH effect. This is not the same as wave vortices depicting OAM. Irrespective of topological charge wave vortices generally have a preferred direction of propagation. However, in TBH the spins switch between CW and CCW continuously over time. This phenomenon inherently satisfies the anti-unitary property of the time-reversal operator.

**Figure 7:**
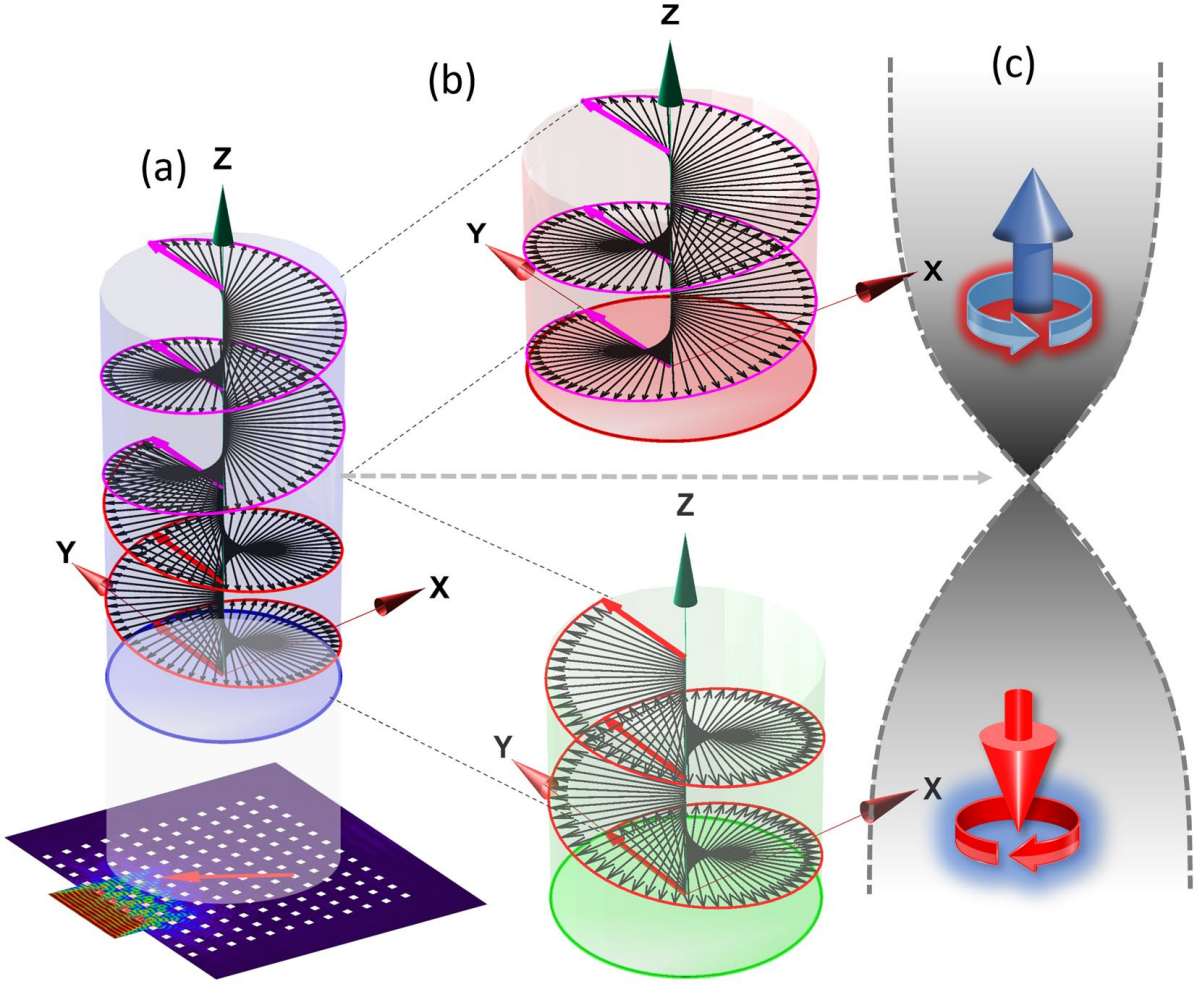
(**a**) TBH yielding polarity phase shift from − 2 π to 2π (0 − 4π) with CW spin (*Spin-Down*), and 2π to − 2π (4π − 8π) with CCW spin (*Spin-Up*) obtained from time dependent study, (**b**) and (**c**) illustrates positive CCW spin and negative CW spin respectively with *Spin-Up* and *Spin-Down* state apparently crossing over the point of inflection over a Möbius strip.

To demonstrate the total global spin event, a segment ($-2\pi to +2\pi$ CW or down spin and $+2\pi to-2\pi$ CCW or up spin) of the total spinning event of $8\pi$ is artistically depicted in Fig. 7a. Like passing over a closed loop *Möbius strip* the CCW up spin flips to CW down spin at $t =\sim 1.77 msec$. With a 2π rotation along the CW direction, the polarity comes back to the initial state after $t = 1.855 msec$. This circular polarization switches spin between positive (CW) to negative (CCW), continuously as if the *spin vector is passing over an Möbius strip* and acquiring a geometric phase. The spin due to circular polarization generates positive SAM. SAM results in shifting the geometric phase from $0$ to $4\pi$ for each full CW rotation and similarly generates negative SAM in shifting the phase from $4\pi$ to $8\pi$. To demonstrate this situation a schematic is shown in Fig. 7b,c. A time axis placed on top of the TBH system with velocity polarity helps understand the polarization over time. The circular polarization is projected to an extent for better understanding the polarity shift over the time in CW direction with a total phase shift of 4π. The initial time was $1.65 msec$ for the arbitrarily selected segment. After the phase change from 0 to 4π, the spin flips at $t =\sim 1.77 msec$. Next, the ${\theta }_{g}(t)$ shift backward, by rotating CCW and record a total phase change from 4π to 0, at $t = 1.92 msec$. It’s like the resultant shift of polarity and its path takes 2 spiral stairs rotating CW ($0-4\pi$) going down and 2 spiral stairs rotating CCW ($4\pi -8\pi$ or $4\pi -0$) going up connected to each other at the phase of 4π on a manifold, and continuously repeating itself as shown in Fig. 7c. That is how the wave energy forms a localized energy sink/whale, keeping the edge preserved. This unique SAM with zero resultant phase change helps containment of the wave energy within the periodic bulk media. And that is how, Topological Blackhole phenomena emerged.

## Conclusion

A unique acoustic phenomenon, termed as topological blackhole, is discovered. The ability of gathering all generated pressure wave energy inside periodic materials makes TBH unique. The containment of energy in the bulk of the PnCs without minimal leakage through the edge state makes it a counter phenomenon of topological insulators. This TBH is a dispersion band dependent phenomenon, and a unique state of deaf band degenerated with a syncline top band, regulates the TBH behavior. Tailoring the PnCs unit cell and resonator size and shape does not tend to affect the generation of the bulk harmonic modes of energy if abnormal polarity condition between the deaf band and the top band is satisfied. Both frequency dependent and time dependent numerical studies have proved that spin dependent topology is the reason behind such a unique state. Due to local and global SAM inside the PnCs arrays the geometric phase shifts from 0 to 2π and then returns to 0 with counter SAM. A map of SAM density is plotted to visualize this effect.

### Supplementary Information

Supplementary Information.

## Data Availability

The data that support the findings of this study are available from the corresponding author upon reasonable request.
