# Supplementary material for: Spin resolved topological bulk state in acoustics
Source: Sci Rep. 2024 Feb 8;14:3213. doi: 10.1038/s41598-024-53226-6 (PMC10853175; doi:10.1038/s41598-024-53226-6)
Supplement: Supplementary file 1 — Supplementary Information. [file 41598_2024_53226_MOESM1_ESM.docx]

S: Supplementary Materials

Spin Resolved Topological Bulk State in Acoustics

**S.1: Phononic Crystal Model: Mathematical Equations for the Band Diagram**

## Generalized Wave Equation of a Periodic Media: Plane wave with in-plane polarity

The in-plane polarity is expressed by the displacement of the media along the $x_{1}$ direction as $u_{1}$ and along the $x_{2}$ direction as $u_{2}$, when the wave propagation is described on the $x_{1}-x_{2}$ plane. Based on the definition of in-plane polarity, the displacements $u_{1}$ and $u_{2}$can be written as a superposition of the Bloch wave modes with periodicity along the $x_{1}$ and $x_{2}$ direction as follows

$u_{1}=\sum_{\mathbf{G}} u_{1}\left( \mathbf{G} \right)e^{i\mathbf{k}\cdot x}e^{-i\omega t}=\sum_{G_{m}} \sum_{G_{n}} A_{mn}e^{i\left( \mathbf{k+G} \right).\mathbf{x}}e^{-i\omega t}=\sum_{G_{m}} \sum_{G_{n}} A_{mn}e^{i\left( k_{1}x_{1}+k_{2}x_{2} \right)}e^{i\left( G_{m}x_{1}+G_{n}x_{2} \right)}$

(S.1)

$u_{2}=\sum_{\mathbf{G}} u_{2}\left( \mathbf{G} \right)e^{i\mathbf{k}\cdot x}e^{-i\omega t}=\sum_{G_{m}} \sum_{G_{n}} A_{mn}e^{i\left( \mathbf{k+G} \right).\mathbf{x}}e^{-i\omega t}=\sum_{G_{m}} \sum_{G_{n}} B_{mn}e^{i\left( k_{1}x_{1}+k_{2}x_{2} \right)}e^{i\left( G_{m}x_{1}+G_{n}x_{2} \right)}$

(S.2)

There are two sets of unknown coefficients, $A_{mn}$ and $B_{mn}$. Two different sets of coefficients for two displacements considered. $\mathbf{G}$ is the Bloch wave vector and $\mathbf{k}$ is the wave vector on the $x_{1}-x_{2}$ plane. Substituting displacement function into the constitutive equation and then in to the governing wave equation will result

$\sum_{\tilde{\mathbf{G}}\mathbf{-G}} \sum_{\mathbf{G}} \left[ \left[ C_{11}^{\tilde{\mathbf{G}}\mathbf{-G}}\left( k_{1}+G_{m} \right)\left( k_{1}+\tilde{G}_{\mathcal{l}} \right)+C_{66}^{\tilde{\mathbf{G}}\mathbf{-G}}\left( k_{2}+G_{n} \right)\left( k_{2}+\tilde{G}_{h} \right) \right] \right.A_{mn}+\left. \left[ C_{12}^{\tilde{\mathbf{G}}\mathbf{-G}}\left( k_{2}+G_{n} \right)\left( k_{1}+\tilde{G}_{\mathcal{l}} \right)+C_{66}^{\tilde{\mathbf{G}}\mathbf{-G}}\left( k_{1}+G_{m} \right)\left( k_{2}+\tilde{G}_{h} \right) \right]B_{mn} \right]e^{i\left( \mathbf{k+}\tilde{\mathbf{G}} \right)\boldsymbol{\cdot x}}=\omega^{2}\sum_{\tilde{\mathbf{G}}\mathbf{-G}} \sum_{\mathbf{G}} \rho_{\left( \tilde{\mathbf{G}}-\mathbf{G} \right)}A_{mn}e^{i\left( \mathbf{k+}\tilde{\mathbf{G}} \right)\boldsymbol{\cdot x}}$ (S.3)

$\sum_{\tilde{\mathbf{G}}\mathbf{-G}} \sum_{\mathbf{G}} \left[ \left[ C_{21}^{\tilde{\mathbf{G}}\mathbf{-G}}\left( k_{1}+G_{m} \right)\left( k_{2}+\tilde{G}_{h} \right)+C_{66}^{\tilde{\mathbf{G}}\mathbf{-G}}\left( k_{2}+G_{n} \right)\left( k_{1}+\tilde{G}_{\mathcal{l}} \right) \right] \right.A_{mn}+\left. \left[ C_{22}^{\tilde{\mathbf{G}}\mathbf{-G}}\left( k_{2}+G_{n} \right)\left( k_{2}+\tilde{G}_{h} \right)+C_{66}^{\tilde{\mathbf{G}}\mathbf{-G}}\left( k_{1}+G_{m} \right)\left( k_{1}+\tilde{G}_{\mathcal{l}} \right) \right]B_{mn} \right]e^{i\left( \mathbf{k+}\tilde{\mathbf{G}} \right)\boldsymbol{\cdot x}}=\omega^{2}\sum_{\tilde{\mathbf{G}}\mathbf{-G}} \sum_{\mathbf{G}} \rho_{\left( \tilde{\mathbf{G}}-\mathbf{G} \right)}B_{mn}e^{i\left( \mathbf{k+}\tilde{\mathbf{G}} \right)\boldsymbol{\cdot x}}$ (S.4)

Where, $C_{ij}$ are the effective material coefficients of the periodic media, $\omega$ is the frequency of the wave. $\rho$ is the effective density of the metamaterial. $\bar{\mathbf{G}}=\bar{G}_{p}{\hat{\mathbf{e}}}_{1}+\bar{G}_{q}{\hat{\mathbf{e}}}_{2}$ , $=G_{m}{\hat{\mathbf{e}}}_{1}+G_{n}{\hat{\mathbf{e}}}_{2}$ & $\mathbf{G}+\bar{\mathbf{G}}=\tilde{\mathbf{G}}$ or $\bar{\mathbf{G}}=\tilde{\mathbf{G}}-\mathbf{G}$ , $\bar{G}_{p}=\left( \tilde{G}_{\mathcal{l}}-G_{m} \right)$ and $\bar{G}_{q}=\left( \tilde{G}_{h}-G_{n} \right)$.

Applying orthogonality condition to Eqs. S.3 and S.4, we get

$\left[ \begin{matrix} S_{11}^{\tilde{\mathbf{G}}\mathbf{-G}} & S_{12}^{\tilde{\mathbf{G}}\mathbf{-G}} \\ S_{21}^{\tilde{\mathbf{G}}\mathbf{-G}} & S_{22}^{\tilde{\mathbf{G}}\mathbf{-G}} \end{matrix} \right]\left\{ \begin{matrix} A_{\mathbf{G}} \\ B_{\mathbf{G}} \end{matrix} \right\}=\omega^{2}\left[ \begin{matrix} \boldsymbol{\rho}_{\left( \tilde{\mathbf{G}}\boldsymbol{-}\mathbf{G} \right)} & \boldsymbol{0} \\ \boldsymbol{0} & \boldsymbol{\rho}_{\left( \tilde{\mathbf{G}}\boldsymbol{-}\mathbf{G} \right)} \end{matrix} \right]\left\{ \begin{matrix} A_{\mathbf{G}} \\ B_{\mathbf{G}} \end{matrix} \right\}$ (S.5)

Where the Components of the elements would be

$S_{11}^{\tilde{\mathbf{G}}\mathbf{-G}}=\left[ C_{11}^{\tilde{\mathbf{G}}\mathbf{-G}}\left( k_{1}+G_{m} \right)\left( k_{1}+\tilde{G}_{\mathcal{l}} \right)+C_{66}^{\tilde{\mathbf{G}}\mathbf{-G}}\left( k_{2}+G_{n} \right)\left( k_{2}+\tilde{G}_{h} \right) \right]$ (S.6)

$S_{12}^{\tilde{\mathbf{G}}\mathbf{-G}}=\left[ C_{12}^{\tilde{\mathbf{G}}\mathbf{-G}}\left( k_{2}+G_{n} \right)\left( k_{1}+\tilde{G}_{\mathcal{l}} \right)+C_{66}^{\tilde{\mathbf{G}}\mathbf{-G}}\left( k_{1}+G_{m} \right)\left( k_{2}+\tilde{G}_{h} \right) \right]$ (S.7)

$S_{21}^{\tilde{\mathbf{G}}\mathbf{-G}}=\left[ C_{21}^{\tilde{\mathbf{G}}\mathbf{-G}}\left( k_{1}+G_{m} \right)\left( k_{2}+\tilde{G}_{h} \right)+C_{66}^{\tilde{\mathbf{G}}\mathbf{-G}}\left( k_{2}+G_{n} \right)\left( k_{1}+\tilde{G}_{\mathcal{l}} \right) \right]$ (S.8)

$S_{22}^{\tilde{\mathbf{G}}\mathbf{-G}}=\left[ C_{22}^{\tilde{\mathbf{G}}\mathbf{-G}}\left( k_{2}+G_{n} \right)\left( k_{2}+\tilde{G}_{h} \right)+C_{66}^{\tilde{\mathbf{G}}\mathbf{-G}}\left( k_{1}+G_{m} \right)\left( k_{1}+\tilde{G}_{\mathcal{l}} \right) \right]$ (S.9)

Here to expand the matrix and visualize how to solve the eigen value problem, first the number of Bloch wave vectors should be assumed. Let’s assume $m=-1, 0 ,+1$ and $n=-1, 0 ,+1$. As $A_{mn}$ is a two indices coefficient it will generate nine coefficients. Similarly, $B_{mn}$ will result another nine coefficients. Thus, the size of the matrices in Eq. S.5 would be $18\times18$ for $m=-1, 0 ,+1$ and $n=-1, 0 ,+1$. For any arbitrary Bloch wave number $N$ the size of the matrices would be $2\left( 2N+1 \right)^{2} \times2\left( 2N+1 \right)^{2}$.

## Circular phononic crystal (Cr) in a host matrix

For circular phononic crystal (Cr) in a host matrix the filling fraction $F_{f}=\frac{\pi{r_{0}}^{2}}{a^{2}}$ (refer Fig. S.1)

- $C_{ij}$ constant

$C_{ij}^{\tilde{\mathbf{G}}\mathbf{-G}}=C_{ij}^{\bar{\mathbf{G}}}=C_{ij}^{pq}=\left( C_{ij} \right)_{I} F_{f}+\left( 1-F_{f} \right)\left( C_{ij} \right)_{II}$ when $\tilde{\mathbf{G}}=\mathbf{G}$ or $\bar{\mathbf{G}}\boldsymbol{=0}$ (S.10)

$C_{ij}^{\tilde{\mathbf{G}}\mathbf{-G}}=C_{ij}^{\bar{\mathbf{G}}}=C_{ij}^{pq}=\left( \left( C_{ij} \right)_{I}-\left( C_{ij} \right)_{II} \right)\frac{2F_{f}J_{1}\left( G_{pq}r_{0} \right)}{G_{pq}r_{0}}$ when $\tilde{\mathbf{G}}\neq\mathbf{G}$ or $\bar{\mathbf{G}}\boldsymbol{\neq0}$ (S.11)

- For Density

$\rho_{\left( \tilde{\mathbf{G}}-\mathbf{G} \right)}=\rho_{\left( \bar{\mathbf{G}} \right)}=\rho_{pq}=\rho_{I}F_{f}+\left( 1-F_{f} \right)\rho_{II}$ when $\tilde{\mathbf{G}}=\mathbf{G}$ or $\bar{\mathbf{G}}\boldsymbol{=0}$ (S.12)

$\rho_{\left( \tilde{\mathbf{G}}-\mathbf{G} \right)}=\rho_{\left( \bar{\mathbf{G}} \right)}=\rho_{pq}=\left( \rho_{I}-\rho_{II} \right)\frac{2F_{f}J_{1}\left( G_{pq}r_{0} \right)}{G_{pq}r_{0}}$ when $\tilde{\mathbf{G}}\neq\mathbf{G}$ or $\bar{\mathbf{G}}\boldsymbol{\neq0}$ (S.13)

## Square phononic crystal (Sq) in a host matrix

For a square or rectangular phononic crystal (Sq) in a host matrix with filling fraction $F_{f}=\frac{b_{1}b_{2}}{a_{1}a_{2}}$ (refer Fig. S.2)

- $C_{ij}$ constant

$C_{ij}^{\tilde{\mathbf{G}}\mathbf{-G}}=C_{ij}^{\bar{\mathbf{G}}}=C_{ij}^{pq}=\left( C_{ij} \right)_{I} F_{f}+\left( 1-F_{f} \right)\left( C_{ij} \right)_{II}$ when $\tilde{\mathbf{G}}=\mathbf{G}$ or $\bar{\mathbf{G}}\boldsymbol{=0}$ (S.14)

$C_{ij}^{\tilde{\mathbf{G}}\mathbf{-G}}=C_{ij}^{\bar{\mathbf{G}}}=C_{ij}^{pq}=\left( \left( C_{ij} \right)_{I}-\left( C_{ij} \right)_{II} \right)F_{f}\left[ \frac{\sin\left( G_{p}\frac{b_{1}}{2} \right)}{G_{p}\left( \frac{b_{1}}{2} \right)} \right]\left[ \frac{\sin\left( G_{q}\frac{b_{2}}{2} \right)}{G_{q}\left( \frac{b_{2}}{2} \right)} \right]$ when $\tilde{\mathbf{G}}\neq\mathbf{G}$ or $\bar{\mathbf{G}}\boldsymbol{\neq0}$ (S.15)


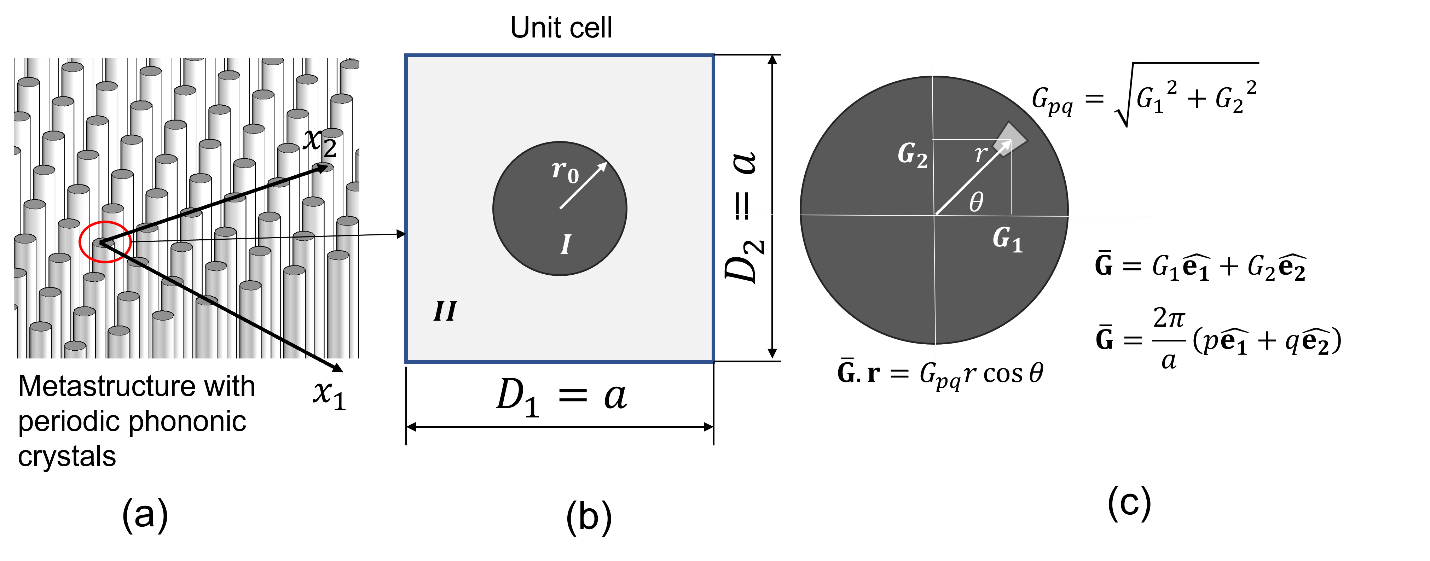


**Figure S.1: a) Circular inclusion in a host matrix b) unit cell, c) Bloch wave vector in polar coordinate**

**
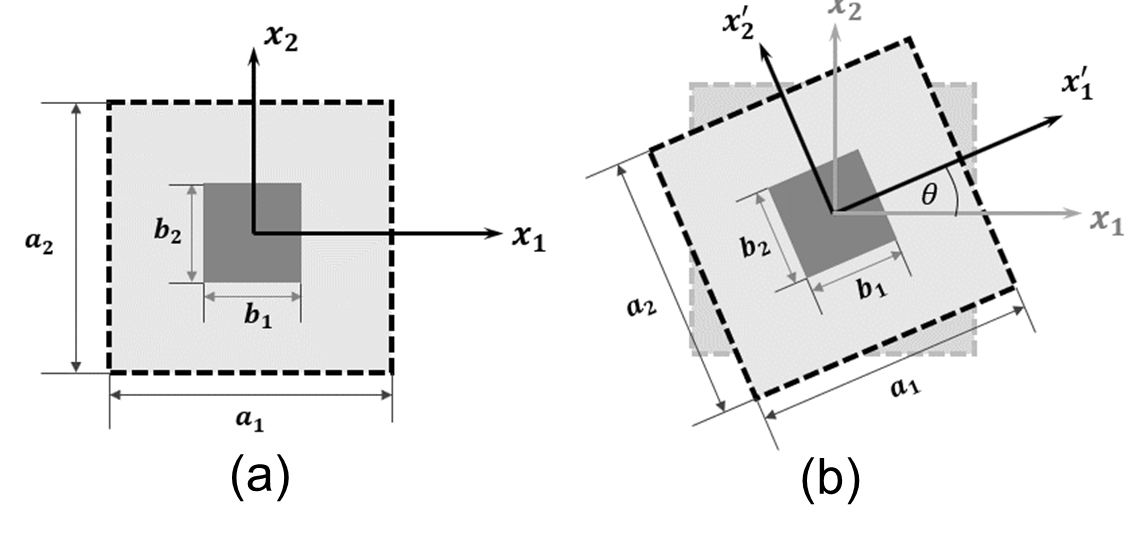
**

**Figure S.2: a) Square inclusion in a host matrix without rotation b) Square inclusion in a host matrix with rotation.**

**S.2 Identification of Topological Phenomena and pattern recognition for explanation**

**Notation:**

SqNR=Square PnCs without Rotation

SqWR=Square PnCs with Rotation

T=Top band

D=Deaf band

B=Bottom band.

- Cr Pristine State $F_{f}$= 0.1169: r = 0.193a – RA: T–D+B: $dc\omega=\sim12.4 kHz$
- Cr Pristine State $F_{f}$ = 0.1169: r = 0.193a – RB: T+D–B: $dc\omega=\sim18.5 kHz$
- Sq Pristine State f$F_{f}$= 0.1169: b = 0.342a – RA: T+D–B: $dc\omega=\sim12.4 kHz$
- Sq Pristine State $F_{f}$ = 0.1169: b = 0.342a – RB: T+D+B: $dc\omega=\sim18.5 kHz$
- Cr: RB: TBH persist between 0.0623 < $F_{f}$ < 0.5: T+D–B satisfied. $dc\omega\uparrow$
- Single mode TBH between 0.0623 < $F_{f}$ < 0.2: T+D–B satisfied.
- SqNR: RA: TBH persist: T+D–B satisfied: narrow band of 0.1024 < $F_{f}$ < 0.1169.
- SqNR: RB: TBH persist: T+D+B satisfied: between a band of 0.09 < $F_{f}$ < 0.16.
- SqWR: Sq Pristine: $F_{f}$ = 0.1169: RA: TBH persist: T+D–B satisfied: 0<$\theta<{\pm17}^{o}$
- SqWR: Sq Pristine: $F_{f}$ = 0.1169: RB: TBH persist: T+D+B satisfied: 0< $\theta<{\pm7}^{o}$
- Circle PnCs (Cr): Filling Fraction 0.1412 – with R=0.212a (a condition for Region A Dirac Cone) But at Region B: Top Band and Deaf Band are degenerated gives TBH.
- Square PnCs (Sq): Filling Fraction with 0.1412 will result b=0.375a in Square PnCs, does not result TBH for Region A: because bottom band is degenerated with the deaf band (A no go for TBH). But at Region B: b=0.375a, Top Band and Deaf Band are degenerated gives TBH
- $F_{f}$= 0.1412: So, Region B for both cases – Sq or Cr PnCs gives TBH.
- Cr: Sq: In both cases T+D is also degenerated at M point. This case only persists for RBs. This is unique because the M point degeneration frequency is above the frequency of TBH phenomena.
- Independent of the direction of excitation of the wave.

**S3: Topologically robustness of TBH against symmetry breaking (Rotation of Sq crystals)**

Rotation of the Cr crystals could not break the symmetry, whereas Sq crystals break the symmetry. Different degrees of symmetry breaking with rotation of the Sq crystals up to 15^o^ were investigated. It was found the TBH is robust against the breaking symmetry where the condition for TBH discussed in the main manuscript is satisfied. Figure S3 shows the results up to 10^o^.


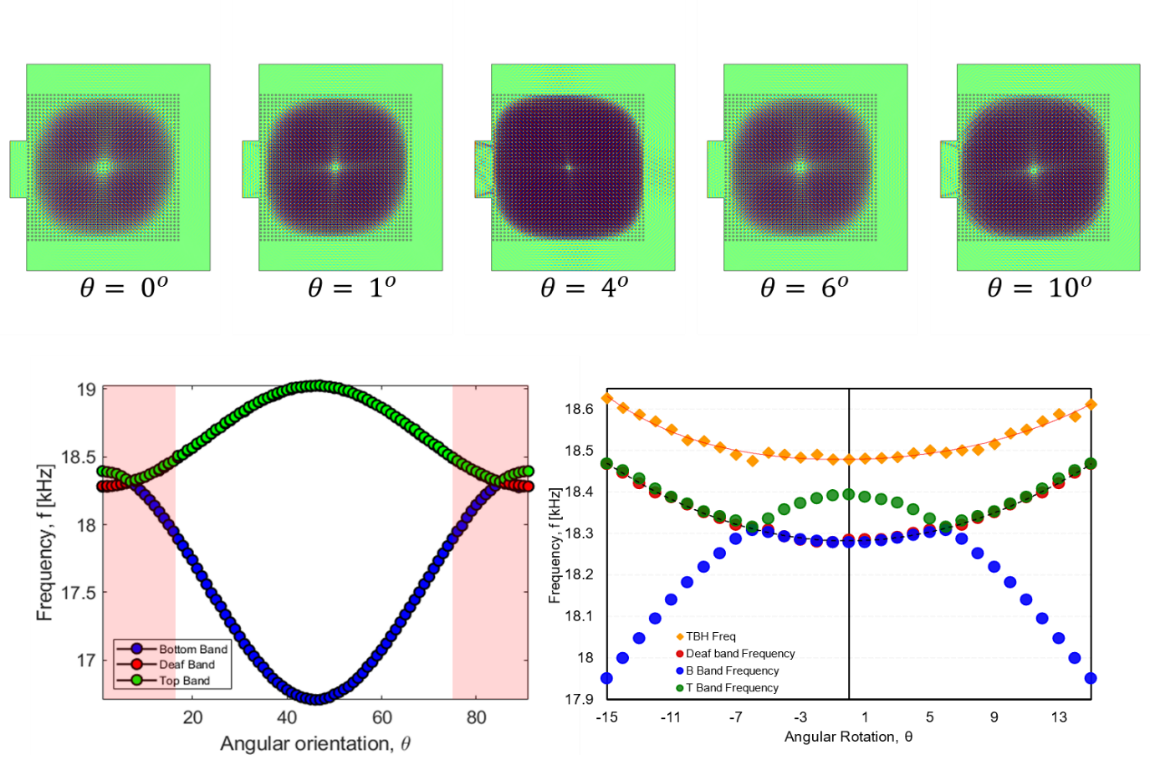


**Figure S.3: a) TBH robustness against symmetry breaking with different degrees of rotation of the Sq crystals, b) shows how the Top, Deaf and Bottom bands take the eigen values with respect to the rotation of the Sq crystals, c) Frequency of occurrence of the TBH phenomena, with respect to the Top, Deaf and Bottom band.**

**S4: Two geometric Configuration to study local Spin Angular Momentum**

**
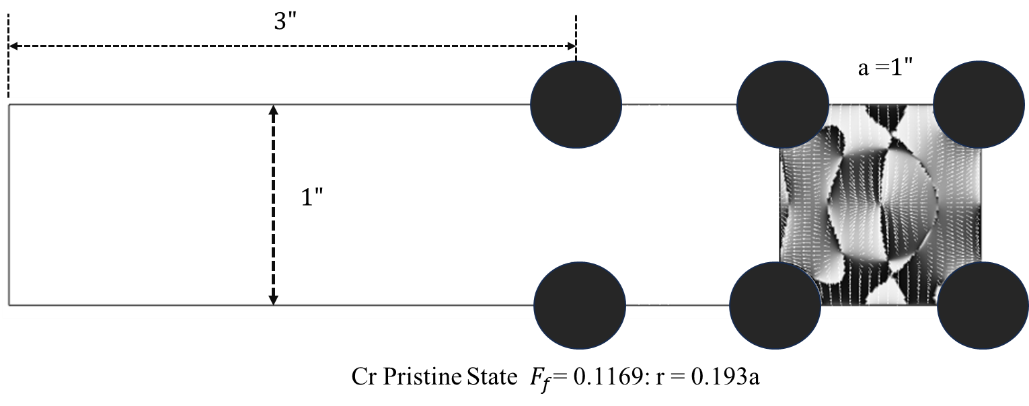
**

**
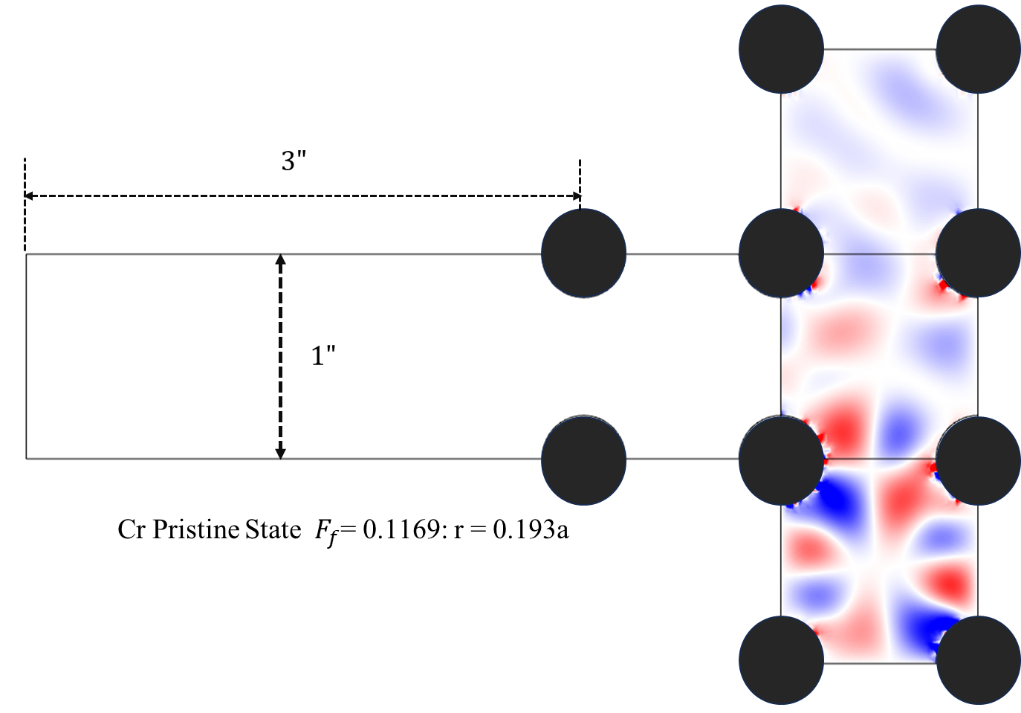
**

**Figure S.4: Schematic showing the structure used for exploring the local spin.**

**S5: Implementation of Spin Angular Momentum calculation in COMSOL.**

$\mathbf{v}\left( x_{j},\omega\right)=[v_{1}(x_{j},\omega)$ $v_{1}(x_{j},\omega)$] ; $s(x_{j},\omega) = \frac{\rho}{2\omega}\left\langle\mathbf{v}\left| \boldsymbol{\sigma} \right|\mathbf{v}^{\boldsymbol{T}} \right\rangle$

imag(i*(acpr.v_tx*acpr.v_ty)+i*(acpr.v_tx*acpr.v_ty))*(acpr.rho/2*freq)

**S6: Global Spin Angular Momentum at TBH**

In frequency analysis at TBH frequencies, the equation to calculate SAM in (Eq. 3 in main document) was implemented and the right column of the figures in Figure S5 is generated.

**
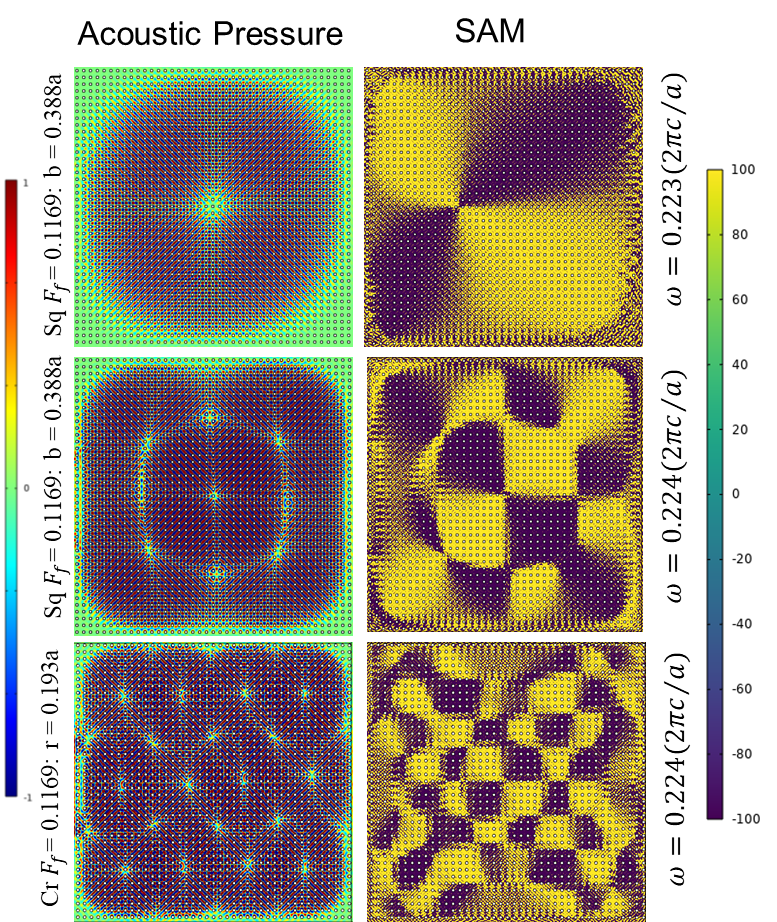
**

**Figure S.5: Global SAM density distribution at different TBH frequencies.**
